# Summarizing the Effective Herbs for the Treatment of Hypertensive Nephropathy by Complex Network and Machine Learning

**DOI:** 10.1155/2021/5590743

**Published:** 2021-06-11

**Authors:** Jia-Ming Huan, Wen-Ge Su, Wei Li, Chao Gao, Peng Zhou, Chun-Sheng Fu, Xiao-Feng Wang, Yi-Min Wang, Yi-Fei Wang

**Affiliations:** ^1^School of Traditional Chinese Medicine, Shandong University of Traditional Chinese Medicine, Jinan 250014, China; ^2^The Affiliated Hospital of Shandong University of Traditional Chinese Medicine, Jinan 250014, China; ^3^First School of Clinical Medicine, Shandong University of Traditional Chinese Medicine, Jinan 250014, China

## Abstract

Hypertensive nephropathy is a common complication of hypertension. Traditional Chinese medicine has been used in the clinical treatment of hypertensive nephropathy for a long time, but the commonly used prescriptions have not been summarized, and the basic therapeutic approaches have not been discussed. Based on data from 3 years of electronic medical records of traditional Chinese medicine used at the Affiliated Hospital of Shandong University of Traditional Chinese Medicine, a complex network and machine learning algorithm was used to explore the prescribed herbs of traditional Chinese medicine in the treatment of hypertensive nephropathy (HN). In this study, complex network algorithms were used to describe traditional Chinese medicine prescriptions for HN treatment. The Apriori algorithm was used to analyze the compatibility of these treatments with modern medicine. Data on the targets and regulatory genes related to hypertensive nephropathy and the herbs that affect their expression were obtained from public databases, and then, the signaling pathways enriched with these genes were identified on the basis of their participation in biological processes. A clustering algorithm was used to analyze the therapeutic pathways at multiple levels. A total of 1499 prescriptions of traditional Chinese medicines used for the treatment of hypertensive renal damage were identified. Fourteen herbs used to treat hypertensive nephropathy act through different biological pathways: huangqi, danshen, dangshen, fuling, baizhu, danggui, chenpi, banxia, gancao, qumai, cheqianzi, ezhu, qianshi, and niuxi. We found the formulae of these herbs and observed that they could downregulate the expression of inflammatory cytokines such as TNF, IL1B, and IL6 and the NF-*κ*B and MAPK signaling pathways to reduce the renal inflammatory damage caused by excessive activation of RAAS. In addition, these herbs could facilitate the deceleration in the decline of renal function and relieve the symptoms of hypertensive nephropathy. In this study, the traditional Chinese medicine approach for treating hypertensive renal damage is summarized and effective treatment prescriptions were identified and analyzed. Data mining technology provided a feasible method for the collation and extraction of traditional Chinese medicine prescription data and provided an objective and reliable tool for use in determining the TCM treatments of hypertensive nephropathy.

## 1. Introduction

HN caused by hypertension commonly damages the kidneys [1]. The International Cross-Sectional Study [2] of Chronic Kidney Disease (CKD) published by the International Society of Nephrology Kidney Disease Data Center (ISN-KDDC) in 2016 showed that hypertension significantly increases the prevalence of CKD (corrected odds ratio (OR) 1.68, 95% confidence interval (CI) 1.60–1.76). The blood pressure control goal of HN remains the focus of discussions. According to the current guidelines [1, 3], the target blood pressure of CKD patients is set to less than 130/80 mmHg, but there is not enough strong evidence from controlled randomized trials of nondiabetic nephropathy to support this view [4]. The African-American Study of Kidney Disease and Hypertension (AASK) showed that lower blood pressure targets do not confer greater benefit than conventional 140/90 mmHg targets. The Ramipril Efficacy in Nephropathy (REIN-2) trial also showed that intensive hypotension does not decrease until end-stage renal disease (ESRD). The Modification of Diet in Renal Disease Study (MDRD) did not address the end point of deterioration of renal function, such as ESRD, and there was a difference in the proportion of angiotensin-converting-enzyme (ACE) inhibitor use between the lower blood pressure group and the usual care group of patients, which limited the observations of the results [5].

Glomerulosclerosis is the most common pathological change associated with hypertension. Long-term hypertension leads to thickening of the renal artery intima, hyaline changes in afferent glomerular arterioles and interlobar arterioles, increased renal vascular resistance, and induced inflammatory reactions and renal tubulointerstitial fibrosis at the same time [6]. First, patients show increased microalbuminuria. After ischemia is aggravated and there is glomerulotubular imbalance, proteinuria, increased serum creatinine levels and urination, loss of appetite, headache, and other signs and symptoms sequentially appear. Albumin excretion greater than 30–299 mg/d or 20–200 *μ*g/min is defined as microalbuminuria (MA), reflecting glomerular endothelial dysfunction and declining nephron filtration function, which is the main marker of HN [4, 7, 8]. Reducing proteinuria is a therapeutic goal in HN [6]. Data from randomized controlled trials showed that lowering blood pressure while inhibiting renin-angiotensin-aldosterone system (RAAS) reduced the severity of proteinuria, showing an additional benefit compared with only a reduction in blood pressure in patients with advanced nephropathy and albuminuria with albumin excretion levels greater than 33.9 mg/mmol [9]. An increase in proteinuria was significantly associated with poor cardiovascular and renal outcomes (adjusted hazard ratio (HR) 1.40, 95% CI 1.11–1.78) [10].

In HN cases, complementary therapies can be good choices for timely intervention and protection against renal damage in the early stage of HN. As a supplementary means of modern medicine, traditional Chinese medicine (TCM) has received increasing attention, and it is one of the most widely used complementary therapies in the world. TCM has clear advantages in the treatment of HN [11–14]; TCM formulae are enriched with active components and are directed toward a variety of targets; therefore, their regulation of a disease network is complex. TCM herbs act on the interaction nodes in the disease biomolecule network, affect component expression or activity, and regulate the imbalance in the disease network [15]. HN involves a number of complex pathological changes; therefore, patients present with a variety of symptom and sign combinations.

In this study, complex networks were used to collect relevant information about TCM prescriptions and disease symptoms from electronic medical records (EMRs) and biological databases, which were used to analyze the prescription network as generated by doctors using herbs in the real world, discuss the topological characteristics of the network, and find the core nodes and connection characteristics of the network, which can be used to mitigate the difficulty posed by the diverse clinical manifestations of this disease. This approach allows the rapid and accurate identification of the common herb combinations used for HN and other diseases and specific symptoms by making efficient use of real-world data [16–18]. When combined with machine learning algorithms, the potential relationships between herbs and diseases as identified in the databases can be used to evaluate and predict the efficacy of traditional Chinese medicine and to infer its mechanisms of action. Previously, the effective herb combinations were condensed, and the disease phenotype and drug chemical properties were integrated at the levels of pharmacology and protein interaction to show the effect of TCM formulae on disease biomolecule networks and to explain biomolecule action mechanisms [19].

Currently, specific prescriptions for the treatment of HN are lacking. Under real-world conditions, the advantages and characteristics of syndrome differentiation for use of TCM treatment can be fully implemented [20] to obtain effective herbal combinations for the treatment of HN and to analyze the mechanism of these herbal combinations in relieving symptoms, protecting the kidneys, and lowering blood pressure. We used TCM EMRs of 1499 prescriptions to mine HN treatment TCM formulae. Through the complex network analysis of these EMRs, 14 herbs used in the treatment of HN were identified. Then, the compatibility of these medicinal herbs was analyzed by the Apriori algorithm, and a clustering algorithm was used to analyze the herbs on the basis of symptoms, targets, and signaling pathway enrichment to determine their therapeutic effects.

## 2. Materials and Methods

### 2.1. Herbs for the Treatment of HN

#### 2.1.1. Setting and Preprocessing of the EMR Data

We collected the EMRs of 30695 anonymous patients diagnosed with hypertension at the Affiliated Hospital of Shandong University of Traditional Chinese Medicine between July 1, 2014, and May 31, 2017, including the diagnosis, demographic characteristics (such as age and sex), chief complaint recorded, and formulae in the TCM prescription. We extracted data on 2055 patients diagnosed with HN and used common terms to identify the signs and symptoms of the HN patients on the basis of the chief recorded complaint and manually referred to the NLM Medical Subject Heading (MeSH) database. Combined with its medical history, other related diseases that can lead to hypertension and CKD were deleted, and the quality was controlled by 2 doctors with over 10 years' experience in the treatment of cardiovascular and renal diseases.

#### 2.1.2. Identifying Combinations of Herbs for the Treatment of HN

We extracted a list of herbs from the formulae of HN patients and set up the herb network with herbs as nodes and the occurrence frequency of the use of two herbs in different formulae as weights. Then, we ran the hierarchical network extraction algorithm to extract herb pairs from the weighted herb network on the basis of the degree coefficient *α* = 2.4. The compatibility relationship of the main herb shell was satisfied with the formula

(1) $$\begin{array}{c} \frac{S_{k}}{k^{\alpha }}*\frac{N}{S_{N}}\geq 1, \end{array}$$

where *N* is the number of all herbs in the data, *S*_*N*_ is the frequency set of all herb pairs, and *S*_*k*_ represents the total frequency of the first *K* herb pairs. The main herb combination structure was extracted iteratively for the analysis of multilayer main herb compatibility. The algorithm was run with LiquoRice software [21].

We used the relative risk (RR) to find highly sensitive herbs between HN patient formulae and non-HN patient formulae. We used the HN patient formulae as the exposure group and the non-HN patient formulae as the nonexposure group, and single herbs used in each group were the outcomes.

(2) $$\begin{array}{c} \text{RR}=\frac{n_{ij}/n_{i}}{\left(n_{j}-n_{ij}\right)/\left(N-n_{i}\right)}, \end{array}$$

where *n*_*i*_ is the sum of the frequency of the herbs in HN patient formulae, *n*_*j*_ is the total frequency of herb_*j*_, and *n*_*ij*_ is the frequency of herb_*j*_ in the HN patient formulae. *N* is the frequency of all herbs. We retained the herbs with RR > 1. Then, we established a 2 × 2 joint table based on these data, calculated the chi-square value, and selected the significant herbs (*P* < 0.05) for further study.

### 2.2. Compatibility of the Herbs

We analyzed the compatibility of the herbs by using the HN prescription of association rules, which are often used to find the conditional dependencies between tuples. We used the classical Apriori algorithm [22] to calculate the herbal association with the *arules* package (version 1.6-6) in *R* 3.6.1, where the support degree (Support), confidence (Confidence), and promotion degree (Lift) retain Support > 0.2 and Confidence > 0.5.

### 2.3. Collection of TCM Symptoms of Herbs

Observing the symptoms of the disease is an important link between disease diagnosis and treatment with TCM. Under the guidance of TCM theory, different herbs can be aimed at different TCM symptoms. The SymMap database contains the TCM symptoms corresponding to herbal medicine that have been agreed upon by experts. The TCM symptoms for which these herbs were used to treat HN were retrieved one by one in the SymMap database.

### 2.4. Identification of Compounds and Targets of Herbal Medicine

We identified compounds and targets of the herbs for the treatment of HN from online databases: Traditional Chinese Medicine Systems Pharmacology Database and Analysis Platform (TCMSP) [23], SymMap [24], the Encyclopedia of Traditional Chinese Medicine (ETCM) [25], and published biomedical literature in PubMed and CNKI databases. The compound and target information were unified into a general format by using the PubChem and UniProt databases, respectively. The data were then organized into an herb-target relationship data set for the herbs. The targets of various herbs were introduced into the STRING database. We retained *Homo sapiens* protein-protein interaction network (PPIN) data with a confidence ≥ 0.9 to establish the PPIN, and the MCODE plug-in of Cytoscape was used to determine the core targets of each herbal medicine.

### 2.5. Collection of HN-Related Genes

The HN gene expression data were retrieved from the NCBI GEO database and analyzed by the GEO2R online analysis tool. The GSE99325 data set contained 20 HN samples and 4 control samples. The genes with *Q* < 0.05 and adj. *P* < 0.05 were regarded as differentially expressed genes in HN.

In addition, we used disease terms in several established databases of human disease-related genes, including Online Mendelian Inheritance in Man (OMIM) [26], DisGeNET [27] and Malacards [28] to identify genes related to HN.

### 2.6. Evaluation of the Importance of Compounds

The human biological functions cannot be realized by individual genes but require ubiquitous interactions between different genes. In this study, we combined the compounds and targets of various herbs with the PPIN we had established to create a biological network. The key compounds in the biological network were calculated by restarting the random walk (RWR) algorithm to measure the closeness between each node and seed node. We used HN-related targets as seed nodes, and the restart probability was 0.75 [21]. The RWR operation was carried out to obtain the stability probability *C*^RWR^ of diffusion of the compounds to measure their importance, which was performed with the *pyrwr* package (version 1.0.0) in Python 3.7.5.

### 2.7. Biological Process Analysis

#### 2.7.1. Gene Annotation and Enrichment Analysis

Using the *clusterProfiler* package [29] (version 3.14.3) in *R* 3.6.1 and org.Hs.eg.db (version 3.10.0) for gene annotation, we carried out a KEGG enrichment analysis of HN and related genes affected by various herbal treatments. Based on the hypergeometric distribution, *Q* < 0.05 was considered an indicator of a significantly enriched pathway, and the same method was used for GO enrichment analysis, with *Q* < 0.05 and adj. *P* < 0.05 as the levels indicating significant enrichment. In addition, we introduced each group of genes into Metascape [30] for multigene list meta-analysis, including functional proteomics, gene screening, and metabolomics.

#### 2.7.2. Hierarchical Clustering

Through GO and KEGG enrichment analyses, the direction of each herb's biological function was determined. To systematically evaluate the similarities and differences of each herb's biological function, we carried out hierarchical clustering according to the number of enriched genes in each KEGG signaling pathway profile and the GO terms for each herb. Moreover, we carried out hierarchical clustering of the collected herbal medicine-related symptoms and targets.

Hierarchical clustering is an unsupervised machine learning method. Initially, a single sample was regarded as a class, and the distance between each class was calculated. The data were merged according to specific rules.

*(1. Distance Calculation.* In this study, Euclidean distance *D*_*ij*_ was used to calculate the distance between herbs and TCM symptoms, targets, or biological processes with the *proxy* package (version 0.4-24) in *R*.

(3) $$\begin{array}{c} D_{ij}=\sqrt{{\left(x_{i}-x_{j}\right)}^{2}+{\left(y_{i}-y_{j}\right)}^{2}}. \end{array}$$

*(2) Hierarchical Clustering.* We used the clustering method of Ward, which is based on analysis of variance. If a classification is reasonable, the error sum of squares (ESS) among the same kinds of herbs should be relatively small, and the ESS between classes should be relatively large [31].

(4) $$\begin{array}{c} \text{ESS}=\sum_{i=1}^{n}x_{i}^{2}-\frac{1}{n}{\left(\sum_{i=1}^{n}x_{i}\right)}^{2}. \end{array}$$

Initially, we regarded a single TCM symptom, target, or biological process as a class, calculated the ESS of each pair of classes after merging, determined the combination with the least increase in total ESS after merging, and then iterated the process until all objects formed a large class with the *flexclust* package (version 1.4-0) in *R* 3.6.1.

## 3. Results

### 3.1. Herbs Used for the Treatment of HN

We selected 1449 prescriptions of traditional Chinese medicine from the EMR data of 2055 patients with HN, and these prescriptions included 425 herbs used 35734 times, and each prescription contained, on average, 23.84 ± 3.88 herbs. The core traditional Chinese medicines used in the treatment prescriptions for HN were obtained by hierarchical extraction algorithm: Radix Astragali (huangqi), Radix Salviae liguliobae (danshen), Radix Codonopsis pilosulae (dangshen), Poria (fuling), Rhizoma Atractylodis macrocephalae (baizhu), Radix Angelicae sinensis (danggui), Pericarpium Citri Reticulatae (chenpi), Rhizoma Pinelliae (banxia), and Radix Glycyrrhizae (gancao). After comparing the RR values, herba Dianthi (qumai), Semen Plantaginis (cheqianzi), Rhizoma Curcumae (ezhu), Semen Euryales (qianshi), and Radix Achyranthis bidentatae (niuxi) were determined to be specific traditional Chinese medicines used for the treatment of HN.

### 3.2. Genes Related to HN

**Figure 1:**
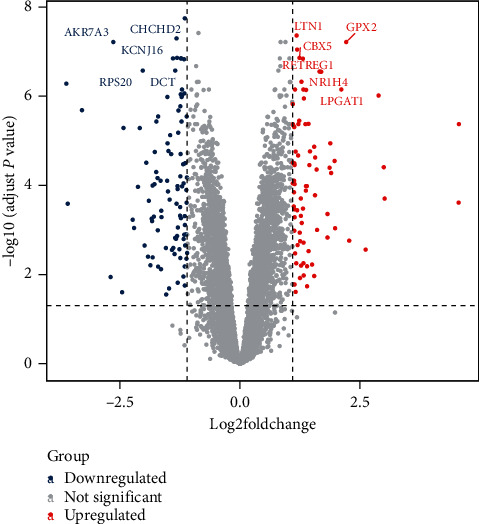
Differentially expressed genes in HN: a blue dot indicates a downregulated gene, and a red dot indicates an upregulated gene.

We obtained 179 differentially expressed genes related to HN from the GSE99325 data set. As shown in Figure 1, the main upregulated genes were LTN1, GPX2, COL4A5, CBX5, MTMR6, RETREG1, NR1H4, RCOR1, LPGAT1, and IMPA1, and downregulated were CHCHD2, KCNJ16, AKR7A3, SIVA1, BARD1, BCAT1, RPL23, AMH, DCT, and RPS20. In addition, we obtained 43 HN genes from the OMIM, DisGeNET, and Malacards databases, and a total of 219 HN-related genes were identified.

### 3.3. Compatibility of Herbs

**Figure 2:**
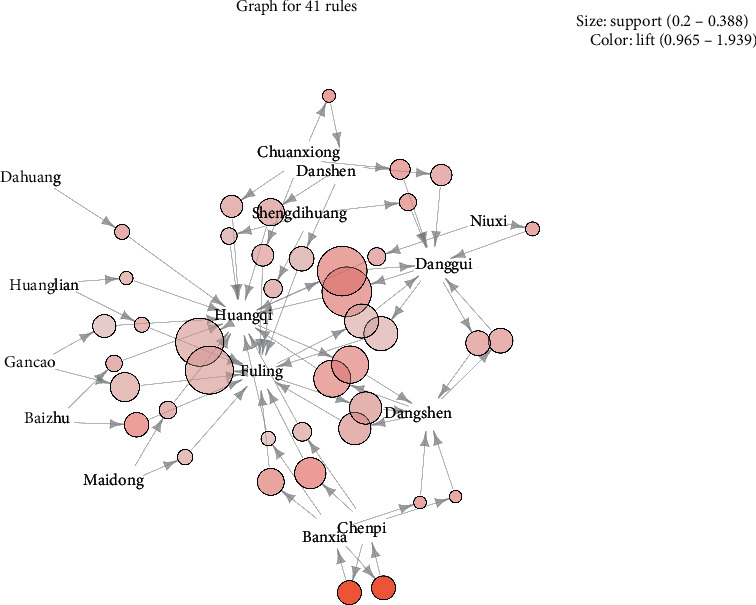
Main rules in the Apriori algorithm: the size of the dot indicates the effect of the herbs, and the darker the color of the dot, the larger the lift.

We found 41 important association rules, as shown in Figure 2, and 14 herbs are generally compatible with other drugs, and the 10 rules for maximum lift values (Table 1) were related to them. For example, banxia-chenpi, chenpi-fuling, baizhu-fuling, chuanxiong-danshen, danggui-huangqi, banxia-fuling, banxia-dangshen, and danghen-huangqi were found through a visual analysis of all prescriptions based on a simultaneously generated grouping matrix, and K-means clustering was used to classify LHS and RHS into one statistical category. Moreover, although Rhizoma Imperatae (baimaogen), Rehmannia glutinosa (shengdihuang), Ophiopogon japonicus (maidong), and Coptidis Rhizoma (huangliang) are used in low frequency; in general, they were used in combination with the 14 identified traditional Chinese medicines in clinical applications to treat HN.

### 3.4. Multilevel Comparison of the 14 Traditional Chinese Herbs

Fourteen kinds of traditional Chinese herbs are used in the clinical treatment of HN, according to their compatibility characteristics, targets, signal pathways, and other information, as determined using MCODE, hierarchical clustering, and other methods for comparative analysis to determine the potential biological mechanism of herbal medicines in the treatment of HN.

#### 3.4.1. TCM Symptom Level

**Figure 3:**
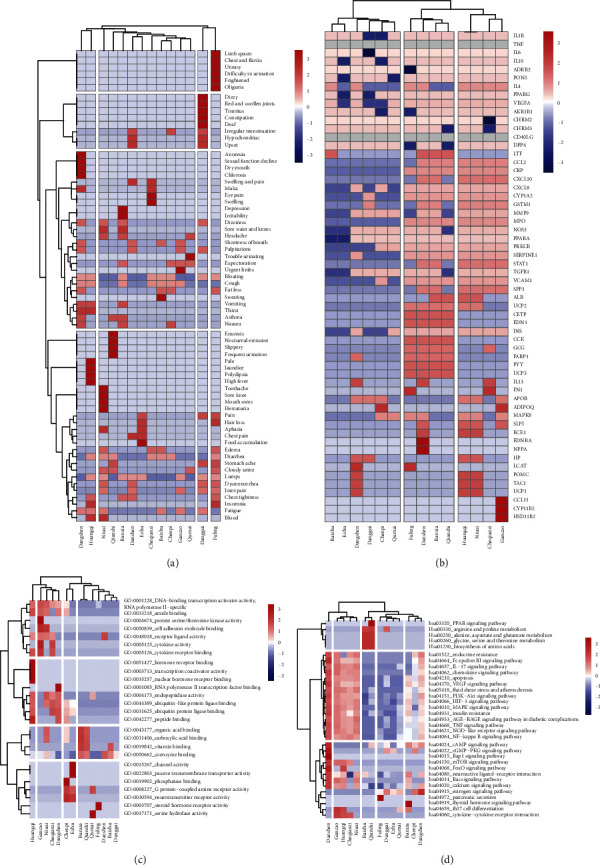
Multilevel comparison of the 14 traditional Chinese herbs. (a) Heat map and hierarchical clustering in target level. (b) Heat map and hierarchical clustering in target level. (c) and (d) Heat map and hierarchical clustering are biological signal pathway results at KEGG signal pathways and GO terms.

Using the SymMap database, we found a total of 68 effective TCM symptoms treated by the 14 herbs, and we established a 14 × 68-dimensional symptom profile in which an herbal medicine that was directed toward a symptom was given a value of 1; otherwise, it was given a value of 0. Then, we used the Ward clustering algorithm for hierarchical clustering in which the similarity between herbs was measured by Euclidean distance.  Figure 3(a) shows that the 14 herbs were divided into 4 groups, and it was found that most herbs could be clustered into one group, and the symptoms include cardiovascular symptoms and humoral metabolic abnormalities such as swelling, dizziness, headache, palpitations, bloating, stomachache, and chest tightness. But individual herbs exhibited obvious effects; for example, fuling can treat the symptoms of body fluid retention caused by renal insufficiency such as difficulty in urination and edema; danggui can treat head discomfort caused by hypertension such as dizzy and tinnitus; and huangqi and dangshen can be used to treat digestive discomfort such as anorexia, thirst, vomiting, and nausea. Overall, the regulation of symptoms by the14 herbs is in line with the common diagnosis and treatments of HN as indicated by TCM theory.

#### 3.4.2. Target Level

After introduction into the STRING database, a total of 58 targets for the 14 herbs were retained, and we established a 14 × 58-dimensional target profile for hierarchical clustering.  Figure 3(b) shows that the 14 herbs were divided into 3 groups. To show more specific biological characteristics of each herbal medicine, we used the MCODE algorithm to identify the core target among the targets.

#### 3.4.3. Pathway Level

**Figure 4:**
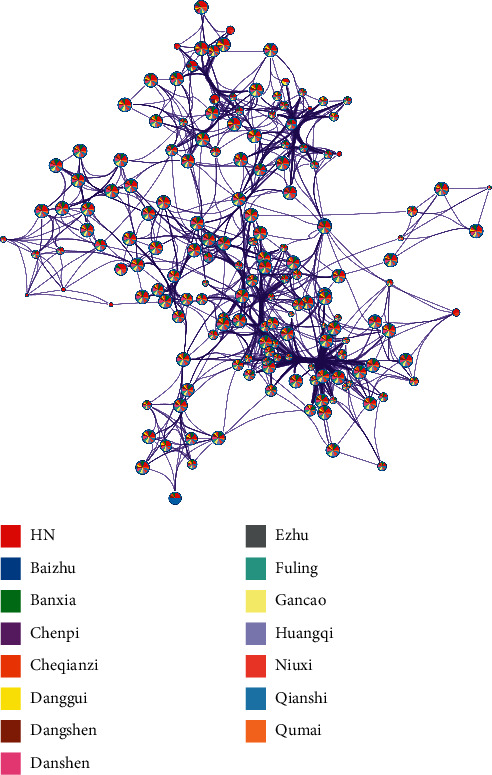
Network of enriched terms represented as pie charts, where pie pieces are color-coded based on the identities of the gene in the herbs and HN. The thicker the line is, the more common the targets of the nodes and the closer their interaction.

**Figure 5:**
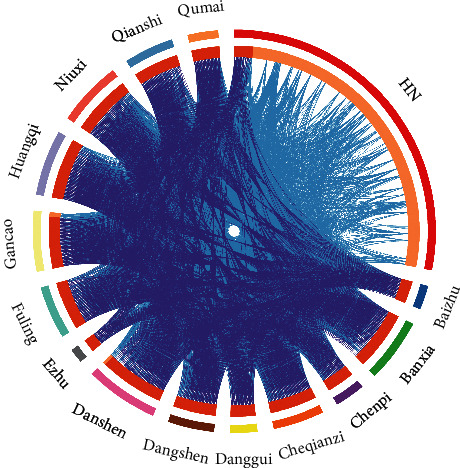
The overlap between the herbs and HN: includes the shared term level, where blue curves link genes that to which the same enriched ontology term is attributed. The inner circle represents gene lists, where hits are arranged along the arc. Genes that were hits in multiple lists are colored in dark orange, and genes unique to a list are shown in light orange.

We analyzed the pathway enrichment of various herbs and HN-related genes and retained the pathways in which the number of enriched genes for each herb treatment in a significantly enriched HN pathway was greater than the quartile of enriched genes expressed upon herbal treatment, and these retained pathways were considered the core pathways for hierarchical clustering. Figures 3(c) and 3(d) show 26 core GO terms, and 33 core KEGG signaling pathways were identified; and 14 × 26-dimensional and 14 × 33-dimensional feather profiles of the core pathways were established. The GO terms and KEGG signaling pathways were divided into 4 categories, and the herbs were divided into 4 categories or 5 categories.  Figure 4 shows that all kinds of herbs have regulatory pathways related to HN.  Figure 5 shows that although the correlation between herbal targets and HN was limited, according to the results of the GO enrichment analysis, a large number of HN-related biological processes could be regulated by these herbs.

#### 3.4.4. Cluster Summary of Herbal Medicines at Different Levels

The table shows that the 14 herbs could be divided into 3 or 4 groups in terms of TCM symptoms, targets, GO enrichment terms, and KEGG enrichment terms (Table 2). The cluster grouping of these 14 herbs was different at different levels, but they could be used to treat HN through various biological pathways.

In terms of TCM symptoms and according to TCM theory, the 14 herbs can be aimed at HN or symptoms related to essential kidney deficiency, such as edema, diarrhea, fatigue, sore waist and knees, fatigue, and diarrhea. Symptoms such as turbid urination, oliguria, and obvious decline of renal function can be specifically treated by fuling.

### 3.5. Identifying Effective Compounds

According to the RWR algorithm, compounds with values in the upper quartile of the RWR were retained as the core effective compounds. Ultimately, 241 effective core compounds were retained, of which the ten compounds with the highest *C*^RWR^ in each group are shown in Table 3. 2-Azaniumylacetate mainly exists in danshen, baizhu, banxia, and qianshi. Quercetin and kaempferol mainly exist in huangqi, danshen, gancao, and niuxi. Myristicin and apigenin mainly exist in dangshen. (2S,3S)-2-Ammonio-3-methylpentanoate and beta-carotene mainly exist in qianshi. Scopoletin was mainly found in baizhu, danggui, and gancao.

## 4. Discussion

Studies have shown that a variety of traditional Chinese medicine prescriptions exhibit clear efficacy in the treatment of HN [11, 12]. Traditional Chinese medicine emphasizes dialectical diagnosis and treatment, but the commonly used treatment with traditional Chinese medicine and its underlying biological basis have not been systematically examined and studied. In this study, 1499 prescriptions of traditional Chinese medicine were identified in EMRs by using complex networks and machine learning algorithms. Fourteen kinds of traditional Chinese medicine were found to be effective in treating HN, and their mechanisms were systematically compared and studied at many levels.

From the perspective of target and pathway enrichment, 14 herbs were involved with 25 core targets, and CHRM2, ADRB2, CXCL8, GCG, TNF, and CXCL10 were the most frequently identified targets. These herbs, particularly danshen, niuxi, cheqianzi, huangqi, and gancao, could act on inflammatory factors, which can be significantly enriched in inflammatory-related pathways such as the HIF-1 signaling pathway, TNF signaling pathway, NOD-like receptor signaling pathway, and NF-*κ*B signaling pathway. These herbs can regulate fibrotic pathways such as the MAPK signaling pathway and PI3K-Akt signaling pathway and vascular endothelial hyperplasia pathways such as the VEGF signaling pathway. Among these herbs, the enrichment of danshen is the most obvious. In addition, danshen can regulate the cAMP signaling pathway, cGMP-PKG signaling pathway, mTOR signaling pathway, Ras signaling pathway, and Rap1 signaling pathway to mediate cell proliferation and differentiation. On the other hand, baizhu and qianshi can participate in the metabolism of amino acids such as glycine, serine, threonine, alanine, aspartate, and glutamate to regulate the cellular processes. Huangqi shows the most extensive regulation cellular life processes, including the regulation of DNA transcription factors and a variety of amino acid metabolic pathways, and it shows a variety of regulatory effects on the function of the kidney and heart. It is suggested that these herbs can produce synergistic and complementary effects in the treatment of HN.

HN is one of the main causes of chronic nephropathy. Arteriosclerosis and hyaline degeneration caused by hypertension are the main pathological changes in HN and are closely related to inflammation and tubulointerstitial fibrosis [6]. Under physiological conditions, the intraglomerular pressure is relatively constant, and when the blood pressure increases, the glomerular arterioles contract properly and reduce the pulse pressure difference. When the self-regulation of renal microcirculation is weakened, the afferent arterioles dilate abnormally, and the intraglomerular pressure increases. At the same time, hypertension and high pulse pressure differences of the large arteries affect the glomerulus, resulting in internal pulsation, stretching, and endothelial injury [32]. These hemodynamic changes activate the RAAS system and aggravate the renal tissue inflammatory response, interstitial fiber hyperplasia, arteriole thickening, and oxidative stress, resulting in impaired nephron function.

The herbs obtained by data mining in this study can inhibit kidney injury with Ang II. The targets of dangfui, niuxi, chenqianzi, huangqi, gancao, dangshen, chenpi, and banxia are significantly related to cAMP signaling pathway, mTOR signaling pathway, and cGMP-PKG signaling pathway. These herbs can inhibit the activity of CHRM1 receptor and GCG on the cell membrane and reduce the activation of cAMP signaling pathway and mTOR signaling pathway, thus inhibiting NF-*κ*B signaling pathway, PI3K-Akt signaling pathway, and other inflammatory-related pathways, degrading secretion of inflammatory factors such as TNF and IL-6. At the same times, it can combat the early renal injury and the inflammatory response caused by Ang II [33], alleviating glomerulosclerosis and epithelial-interstitial transition caused by oxidative stress [34, 35]. Podocyte damage is also a core kidney injury caused by HN. The Ang II receptor was overexpressed in podocytes, which induced the foot process to disappear [36], and vascular endothelial inflammatory factors were upregulated, activating the PI3K/Akt and MAPK signaling pathway to promote vascular endothelial proliferation, thus inducing recombination of the podocyte cytoskeleton [37]. Chenpi, qumai, fuling, gancao, niuxi, danshen, and huangqi can directly or indirectly affect inflammatory factors such as TNF, affect MAPK8 activity, and regulate the MAPK signaling pathway. In addition, chenpi can suppress ADIPOQ and mitigate the phosphorylation levels of AMPK, and the AMPK and mTOR signaling that controls the cytoskeleton was reduced, resulting in decreasing podocyte injury [38].

HN is also a long-term cause of nephron damage. A previous study showed that the pathological renal changes caused by HN were progressive, with glomerular damage appearing first and renal tubular atrophy and interstitial fibrosis appearing as late manifestations in an SHR model [39]. Inflammatory cytokines such as IFN *γ*, IL-6, and TNF overactivated STAT1, STAT3, and NF-*κ*B transcription factors to promote glomerulosclerosis and renal interstitial fibrosis [40–42]. NF-*κ*B/p65 has been identified as the key factor mediating renal damage induced by Ang II [43]. Qianshi, huangqi, dangshen, chenpi, and banxia can act on PRKCB to inhibit the production of NF-*κ*B transcription factors and reduce the renal inflammation and injury induced by Ang II, downregulate the expression levels of TNF, IL-6, and IL-8 in the kidney, and reduce the mRNA and protein levels of type I and IV collagen [44]. According to the enrichment results in this study, qianshi, baizhu, and huangqi can regulate the levels of serine and other amino acids and nuclear factors, slow nephron damage, and protect renal vessels damaged by long-term hypertension.

The therapeutic effects of these herbs on HN are mainly the deceleration of the renal damage caused by hypertension, protection of nephrons and podocytes, reduction in blood pressure and the amount of protein in urine, and slowing renal interstitial fibrosis and maintaining nephron function, but the effect of a single herbal medicine is often limited. To achieve the expected therapeutic effects, these herbs need to be combined. These herbs are often used in the clinic. This finding is consistent with the results in this study, showing that these herbs fall under the important compatibility rules identified by the Apriori algorithm. To some extent, the results of the multilevel analysis indicated the possible effective pathway of these traditional Chinese medicine combinations, which is consistent with the mechanism of renal injury caused by HN, but this study also has many limitations. First, the compound information, targets, and disease-related genes were all identified in open databases, and there is still a lack of experimental studies on proteomics and metabonomics. Second, the effects of these herbs on the NF-*κ*B signaling pathway, cAMP signaling pathway, mTOR signaling pathway, and other pathways need to be further studied.

## 5. Conclusions

Based on the traditional Chinese medicine prescription data of HN treated in the Affiliated Hospital of Shandong University of Traditional Chinese Medicine from 2014 to 2017, traditional Chinese medicine for HN treatment was found by using a complex network algorithm and calculating the relative risk. Fourteen herbs were compared and analyzed at multiple levels with a clustering algorithm. Through the clustering results obtained for different levels of analysis, these herbs were found to play multilevel roles in the biological regulation of HN. This study also showed that the use of a data mining algorithm can be used to summarize the TCM treatment prescription of HN accurately and with high reliability and can provide a direction for clinical practice and future experimental research.

## Figures and Tables

**Table 1:** Ten rules for maximum lift values.

LHS		RHS	Support	Confidence	Coverage	Lift	Count
Chenpi	=>	Banxia	0.258	0.701	0.368	1.939	387
Banxia	=>	Chenpi	0.258	0.714	0.362	1.939	387
Chenpi	=>	Fuling	0.294	0.799	0.368	1.337	441
Baizhu	=>	Fuling	0.261	0.795	0.328	1.330	391
Chuanxiong	=>	Danshen	0.203	0.540	0.376	1.301	304
Danggui	=>	Huangqi	0.388	0.758	0.511	1.285	581
Huangqi	=>	Danggui	0.388	0.656	0.590	1.285	581
Banxia	=>	Fuling	0.273	0.755	0.362	1.262	409
Banxia	=>	Dangshen	0.201	0.555	0.362	1.256	301
Dangshen	=>	Huangqi	0.324	0.733	0.442	1.242	486

**Table 2:** Clusters of the herbs at different levels.

Levels		TCM symptom	Target	GO term	KEGG signal pathway
Clusters	1	Fuling	Gancao, cheqianzi, niuxi, huangqi	Danggui, baizhu, danshen, fuling, qumai, qianshi, banxia	Danshen, chenpi, banxia
	2	Danggui	qianshi, banxia, danshen, fuling	Ezhu, chenpi	Qumai, ezhu, danggui, fuing
	3	Qumai, gancao, chenpi, baizhu, ezhu, danshen, banxia, qianshi, niuxi	Qumai, chenpi, dangui, dangshen, ezhu, baizhu	Dangshen, cheqianzi, niuxi, gancao	Qianshi, baizhu
	4	Huanqi, dangshen	—	Huangqi	Niuxi, cheqianzi, huangqi, gancao

**Table 3:** The top 10 compounds with the highest *C*^RWR^.

PubChem CID	Compound	Formula	RWR
5257127	2-Azaniumylacetate	C_2_H_5_NO_2_	1.82E − 04
5280343	Quercetin	C_15_H_10_O_7_	1.40E − 04
5281708	Daidzein	C_15_H_10_O_4_	1.01E − 04
5280460	Scopoletin	C_10_H_8_O_4_	7.49E − 05
4276	Myristicin	C_11_H_12_O_3_	7.46E − 05
5280443	Apigenin	C_15_H_10_O_5_	6.45E − 05
177	Acetaldehyde	C_2_H_4_O	6.03E − 05
7043901	(2S,3S)-2-Ammonio-3-methylpentanoate	C_6_H_13_NO_2_	5.87E − 05
5280863	Kaempferol	C_15_H_10_O_6_	5.44E − 05
5280489	Beta-carotene	C_40_H_56_	4.30E − 05

## Data Availability

The TCM prescription data used to support the findings of this study are available from the corresponding author upon request.

## Abbreviations

HN: Hypertensive nephropathy
CKD: Chronic kidney disease
ISN-KDDC: The International Society of Nephrology Kidney Disease Data Center
OR: Odds ratio
CI: Confidence interval
AASK: The African-American Study of Kidney Disease and Hypertension
REIN-2: The Ramipril Efficacy in Nephropathy
ESRD: End-stage renal disease
MDRD: The Modification of Diet in Renal Disease Study
ACE: Angiotensin-converting-enzyme
MA: Microalbuminuria
RAAS: Renin-angiotensin-aldosterone system
HR: Hazard ratio
TCM: Traditional Chinese medicine
EMR: Electronic medical record
MeSH: The NLM Medical Subject Heading
RR: Relative risk
TCMSP: Traditional Chinese Medicine Systems Pharmacology Database and Analysis Platform
ETCM: Encyclopedia of Traditional Chinese Medicine
PPIN: Protein-protein interaction network
OMIM: Online Mendelian Inheritance in Man
RWR: Restarting the random walk
ESS: The error sum of squares
huangqi: Radix astragali
danshen: Radix salviae liguliobae
dangshen: Radix codonopsis pilosulae
fuling: Poria
baizhu: Rhizoma atractylodis macrocephalae
danggui: Radix angelicae sinensis
chenpi: Pericarpium citri reticulatae
banxia: Rhizoma pinelliae
gancao: Radix glycyrrhizae
qumai: Herba dianthi
ezhu: Rhizoma curcumae
qianshi: Semen euryales
niuxi: Radix achyranthis bidentatae
baimaogen: Rhizoma imperatae
shengdihuang: Rehmannia glutinosa
maidong: Ophiopogon japonicus
huangliang: Coptidis rhizoma.

## Supplementary Materials

Supplementary Materials. Apriori: 41 important association rules of the 14 herbs obtained for the Apriori algorithm. Core target of herbs: the core target of 14 herbs. cRWR of effective compounds: *C*^RWR^ of 241 effective core compounds. Figure 4 data: pathway enrichment data for the herbs and HN needed to draw Figure 4.

## References

[1] Williams B., Mancia G., Spiering W (2018). ESC/ESH guidelines for the management of arterial hypertension. *European Heart Journal*.

[2] Ene-Iordache B., Perico N., Bikbov B. (2016). Chronic kidney disease and cardiovascular risk in six regions of the world (ISN-KDDC): a cross-sectional study. *The Lancet Global Health*.

[3] Whelton P. K., Carey R. M., Aronow W. S (2017). PCNA guideline for the prevention, detection, evaluation, and management of high blood pressure in adults: a report of the American college of cardiology/American heart association task force on clinical practice guidelines. *Hypertension*.

[4] Hart P. D., Bakris G. L. (2010). Hypertensive nephropathy: prevention and treatment recommendations. *Expert Opinion on Pharmacotherapy*.

[5] Weir M. R. (2014). Hypertension and kidney disease. *Journal of the American Society of Hypertension*.

[6] Seccia T. M., Caroccia B., Calò L. A. (2017). Hypertensive nephropathy moving from classic to emerging pathogenetic mechanisms. *Journal of Hypertension*.

[7] Bakris G. L. (2004). Clinical importance of microalbuminuria in diabetes and hypertension. *Current Hypertension Reports*.

[8] Ibsen H., Olsen M. H., Wachtell K. (2005). Reduction in albuminuria translates to reduction in cardiovascular events in hypertensive patients. *Hypertension*.

[9] Bakris G. L., Sarafidis P. A., Weir M. R. (2010). Renal outcomes with different fixed-dose combination therapies in patients with hypertension at high risk for cardiovascular events (ACCOMPLISH): a prespecified secondary analysis of a randomised controlled trial. *The Lancet*.

[10] Schmieder R. E., Mann J. F. E., Schumacher H. (2011 Jul). Changes in albuminuria predict mortality and morbidity in patients with vascular disease. *Journal of the American Society of Nephrology*.

[11] Dong Y., Yue B., Qian M. (2018). JYYS granule mitigates renal injury in clinic and in spontaneously hypertensive rats by inhibiting NF-*κ*B signaling-mediated microinflammation. *Evidence-based Complementary and Alternative Medicine-eCAM*.

[12] Owoicho Orgah J., Wang M., Yang X (2018). Danhong injection protects against hypertension-induced renal injury via down-regulation of myoglobin expression in spontaneously hypertensive rats. *Kidney and Blood Pressure Research*.

[13] Wu L., Liu M., Fang Z. (2018). Combined therapy of hypertensive nephropathy with breviscapine injection and antihypertensive drugs: a systematic review and a meta-analysis. *Evidence-based Complementary and Alternative Medicine-eCAM*.

[14] Li Y., Yan S., Qian L., Wu L., Zheng Y., Fang Z. (2020). Danhong injection for the treatment of hypertensive nephropathy: a systematic review and meta-analysis. *Frontiers in Pharmacology*.

[15] Zhao J., Jiang P., Zhang W. (2010). Molecular networks for the study of TCM pharmacology. *Briefings in Bioinformatics*.

[16] Yang K., Lu K., Wu Y (2021). A network-based machine-learning framework to identify both functional modules and disease genes. *Human Genetics*.

[17] Wang N., Li P., Hu X. (2019). Herb target prediction based on representation learning of symptom related heterogeneous network. *Computational and Structural Biotechnology Journal*.

[18] Wang N., Du N., Peng Y. (2021). Network patterns of herbal combinations in traditional Chinese clinical prescriptions. *Frontiers in Pharmacology*.

[19] Yang J., Tian S., Zhao J., Zhang W. (2020). Exploring the mechanism of TCM formulae in the treatment of different types of coronary heart disease by network pharmacology and machining learning. *Pharmacological Research*.

[20] Liu B. (2013). The paradigm of clinical scientific research of traditional Chinese medicine in the real world. *Journal of Traditional Chinese Medicine*.

[21] Zhou X., Chen S., Liu B (2010). Development of traditional Chinese medicine clinical data warehouse for medical knowledge discovery and decision support. *Artificial Intelligence in Medicine*.

[22] Srikant R., Vu Q., Agrawal R. Mining association rules with item constraints.

[23] Ru J., Li P., Wang J (2014). TCMSP: a database of systems pharmacology for drug discovery from herbal medicines. *Journal of Cheminformatics*.

[24] Wu Y., Zhang F., Yang K. (2019). SymMap: an integrative database of traditional Chinese medicine enhanced by symptom mapping. *Nucleic Acids Research*.

[25] Xu H.-Y., Zhang Y.-Q., Liu Z.-M. (2019). ETCM: an encyclopaedia of traditional Chinese medicine. *Nucleic Acids Research*.

[26] Amberger J. S., Bocchini C. A., Schiettecatte F., Scott A. F., Hamosh A. (2015). OMIM.org: online mendelian inheritance in man (OMIM®), an online catalog of human genes and genetic disorders. *Nucleic Acids Research*.

[27] Piñero J., Bravo À., Queralt-Rosinach N. (2017). DisGeNET: a comprehensive platform integrating information on human disease-associated genes and variants. *Nucleic Acids Research*.

[28] Rappaport N., Twik M., Plaschkes I. (2017). MalaCards: an amalgamated human disease compendium with diverse clinical and genetic annotation and structured search. *Nucleic Acids Research*.

[29] Yu G., Wang L.-G., Han Y., He Q.-Y. (2012). cluster profiler: an R package for comparing biological themes among gene clusters. *OMICS: A Journal of Integrative Biology*.

[30] Zhou Y., Zhou B., Pache L. (2019). Metascape provides a biologist-oriented resource for the analysis of systems-level datasets. *Nature Communications*.

[31] Ward J. H., Joe H. (1963). Hierarchical grouping to optimize an objective function. *Journal of the American Statistical Association*.

[32] Stompór T., Perkowska-Ptasińska A. (2020). Hypertensive kidney disease: a true epidemic or rare disease?. *Polish Archives of Internal Medicine*.

[33] Coelho S. C., Berillo O., Caillon A. (2018). Three-month endothelial human endothelin-1 overexpression causes blood pressure elevation and vascular and kidney injury. *Hypertension*.

[34] Mennuni S., Rubattu S., Pierelli G., Tocci G., Fofi C., Volpe M. (2014). Hypertension and kidneys: unraveling complex molecular mechanisms underlying hypertensive renal damage. *Journal of Human Hypertension*.

[35] Liu M., Ning X., Li R. (2017). Signalling pathways involved in hypoxia-induced renal fibrosis. *Journal of Cellular and Molecular Medicine*.

[36] Hoffmann S., Podlich D., Hähnel B., Kriz W., Gretz N. (2004). Angiotensin II type 1 receptor overexpression in podocytes induces glomerulosclerosis in transgenic rats. *Journal of the American Society of Nephrology*.

[37] Mocker A., Hilgers K. F., Cordasic N. (2019). Renal chemerin expression is induced in models of hypertensive nephropathy and glomerulonephritis and correlates with markers of inflammation and fibrosis. *International Journal of Molecular Sciences*.

[38] Rinschen M. M., Palygin O., Guijas C (2019). Metabolic rewiring of the hypertensive kidney. *Science Signaling*.

[39] Hatziioanou D., Barkas G., Critselis E. (2018). Chloride intracellular channel 4 overexpression in the proximal tubules of kidneys from the spontaneously hypertensive rat: insight from proteomic analysis. *Nephron*.

[40] O’Brown Z. K., Van Nostrand E. L., Higgins J. P., Kim S. K. (2015). The inflammatory transcription factors NF*κ*B, STAT1 and STAT3 drive age-associated transcriptional changes in the human kidney. *Plos Genetics*.

[41] He P., Li Z., Yue Z. (2018). SIRT3 prevents angiotensin II-induced renal tubular epithelial-mesenchymal transition by ameliorating oxidative stress and mitochondrial dysfunction. *Molecular and Cellular Endocrinology*.

[42] Said E., Zaitone S. A., Eldosoky M., Elsherbiny N. M. (2018). Nifuroxazide, a STAT3 inhibitor, mitigates inflammatory burden and protects against diabetes-induced nephropathy in rats. *Chemico-Biological Interactions*.

[43] Henke N., Schmidt-Ullrich R., Dechend R. (2007). Vascular endothelial cell-specific NF-*κ*B suppression attenuates hypertension-induced renal damage. *Circulation Research*.

[44] Lu Q., Ma Z., Ding Y. (2019). Circulating miR-103a-3p contributes to angiotensin II-induced renal inflammation and fibrosis via a SNRK/NF-*κ*B/p65 regulatory axis. *Nature Communications*.

