# Supplementary material for: Summarizing the Effective Herbs for the Treatment of Hypertensive Nephropathy by Complex Network and Machine Learning
Source: Evid Based Complement Alternat Med. 2021 Jun 11;2021:5590743. doi: 10.1155/2021/5590743 (PMC8214481; doi:10.1155/2021/5590743)
Supplement: Supplementary Materials. — Apriori: 41 important association rules of the 14 herbs obtained for the Apriori algorithm. Core target of herbs: the core target of 14 herbs. cRWR of effective compounds: CRWR of 241 effective core compounds. Figure 4 data: pathway enrichment data for the herbs and HN needed to draw Figure 4. [file 5590743.f1.zip › 5590743.f1/SUPPLEMENTARY DESCRIPTION.docx]

**SUPPLEMENTARY DESCRIPTION**

**Supplementary Material**

**Apriori**: 41 important association rules of the 14 herbs obtained for the Apriori algorithm.

**core target of herbs**: the core target of 14 herbs.

**cRWR of effective compounds**: $C^{RWR}$ of 241 effective core compounds.

**Figure 4 data**: Pathway enrichment data for the herbs and HN needed to draw Figure 4.
